# Chronic-Alcohol-Abuse-Induced Oxidative Stress in the Development of Acute Respiratory Distress Syndrome

**DOI:** 10.1100/2012/740308

**Published:** 2012-12-27

**Authors:** Yan Liang, Samantha M. Yeligar, Lou Ann S. Brown

**Affiliations:** ^1^Division of Neonatal-Perinatal Medicine, Department of Pediatrics, Emory University and Children's Healthcare of Atlanta Center for Developmental Lung Biology, Atlanta, GA 30322, USA; ^2^Department of Medicine, Atlanta Veterans' Affairs and Emory University Medical Centers, Decatur, GA 30033, USA

## Abstract

Chronic alcohol ingestion increases the risk of developing acute respiratory distress syndrome (ARDS), a severe form of acute lung injury, characterized by alveolar epithelial and endothelial barrier disruption and intense inflammation. Alcohol abuse is also associated with a higher incidence of sepsis or pneumonia resulting in a higher rate of admittance to intensive care, longer inpatient stays, higher healthcare costs, and a 2–4 times greater mortality rate. Chronic alcohol ingestion induced severe oxidative stress associated with increased ROS generation, depletion of the critical antioxidant glutathione (GSH), and oxidation of the thiol/disulfide redox potential in the alveolar epithelial lining fluid and exhaled breath condensate. Across intracellular and extracellular GSH pools in alveolar type II cells and alveolar macrophages, chronic alcohol ingestion consistently induced a 40–60 mV oxidation of GSH/GSSG suggesting that the redox potentials of different alveolar GSH pools are in equilibrium. Alcohol-induced GSH depletion or oxidation was associated with impaired functions of alveolar type II cells and alveolar macrophages but could be reversed by restoring GSH pools in the alveolar lining fluid. The aims of this paper are to address the mechanisms for alcohol-induced GSH depletion and oxidation and the subsequent effects in alveolar barrier integrity, modulation of the immune response, and apoptosis.

## 1. Epidemiology of Alcohol Abuse, ARDS, and Lung Injury

Alcohol abuse is defined as the recurring use of alcoholic beverages despite negative consequences [1]. Each year, alcohol abuse costs ~100,000 lives and ~$100 billion in healthcare expenditures in the US [2, 3]. In CDC analysis of 2010 drinking patterns [4], people aged 18–24 years had a higher prevalence (28.2%) and intensity (9.3 drinks/occasion) of alcohol use, but people ≥65 years had a higher frequency (5.5 episodes/month). Although households with incomes <$25,000 had the highest frequency (5.0 episodes/month) and intensity (8.5 drinks/occasion), households with incomes ≥$75,000 had the highest prevalence (20.2%). In addition to increasing the risks of developing an alcohol use disorder, alcohol abuse is problematic and often associated with increased medical risks such as cardiovascular disease, mal-absorption, chronic pancreatitis, alcoholic liver disease, and cancer. The higher incidence of sepsis or pneumonia in subjects that abuse alcohol results in a higher rate of admittance to an intensive care unit [5, 6], longer inpatient stays, higher healthcare costs [7], and a 2–4 times greater mortality rate [8–11]. Although long-term use of alcohol in excessive quantities is capable of affecting every organ system in the body, the investigation of the effects of alcohol on the lung is in its early stage. The most significant pulmonary effects of alcohol abuse are the increased risks of bacterial infection and acute lung injury (ALI).

**Figure 1:**
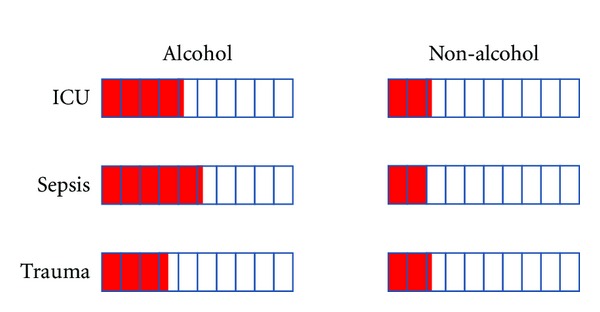
Alcohol abuse increased the incidence of acute respiratory distress syndrome (ARDS) in critically ill patients with an identified at-risk diagnosis. Presented values were adapted from [11]. The red bar indicates the incidence (%) of ARDS when stratified by the at-risk diagnosis and history of alcohol abuse.

Acute respiratory distress syndrome (ARDS) is recognized as the most severe form of acute lung injury, a form of diffuse alveolar injury with bilateral pulmonary infiltrates and severe hypoxemia in the absence of cardiogenic pulmonary edema [12]. Pathologically, ARDS is most commonly associated with diffuse alveolar damage characterized by inflammation of the lung parenchyma leading to impaired gas exchange with concomitant systemic release of inflammatory mediators causing inflammation and hypoxemia. The consequences of ARDS are severe, frequently resulting in multiple organ failure and death. A perspective study conducted in King County, Washington, found that the age-adjusted incidence of ALI was 86.2 per 100,000 person-years [13]. Based on these studies, it is estimated that 190,600 ARDS cases exist in the United States annually and that these cases are associated with a mortality rate of >40% [14, 15]. There are many risk factors for the development of ARDS including sepsis, trauma, pneumonia, hypertransfusion, pancreatitis, surgery, and others [16, 17]. Although these risk factors account for 85% of ARDS, only a minority (about 30%) of these at-risk individuals develop ARDS suggesting the involvement of other risk factors. Recent evidence showed that a history of alcohol abuse is an independent risk factor that increases the odds of any at-risk individual developing ARDS (Figure 1). The first study which identified the effect of alcohol abuse on ARDS found that among 351 critical ill patients, the incidence of ARDS in patients with a history of alcohol abuse was significantly higher than in patients without a history of alcohol abuse (43% versus 22%) [11]. In patients with sepsis as their primary at-risk diagnosis for the development of ARDS, a positive history of chronic alcohol abuse increased the incidence of ARDS by 2-fold when compared to septic patients without a history of chronic alcohol abuse [18]. For trauma patients, the incidence of ARDS is 34% in those with a history of alcohol abuse compared with 22% for trauma patients without such a history [11]. These clinical studies suggest that an alcohol use disorder represents ~50% of all ARDS cases with an average age of 30–35 years [9–11, 19, 20]. Thus, alcohol related lung injury annually contributes to the premature deaths of 30,000–40,000 people in the US, affecting a younger population and rivaling alcohol-mediated liver disease.

## 2. An Alcohol Use Disorder Depletes Glutathione in the Lung

The discovery of alcohol abuse-associated ARDS has led to critical investigations of the underlying mechanisms responsible for increased susceptibility to ALI. Three sentinel effects of chronic alcohol ingestion induced oxidative stress are the increase of reactive oxygen species (ROS) generation, depletion of critical antioxidants, and oxidation of the thiol/disulfide redox potential in the alveolar epithelial lining fluid (ELF) [19]. In the ELF, a history of an alcohol use disorder is associated with an 80% decrease in the critical antioxidant glutathione (GSH). There was a corresponding oxidation of the glutathione pool to the glutathione disulfide form (GSSG) resulting in an ~40 mV oxidation of the GSH/GSSG redox potential (*E*_*h*_), even when controlled for smoking status [21]. The alcohol-induced GSH depletion observed in the ELF was not reflected in plasma where only a 30 mV GSH/GSSG oxidation was observed in subjects who both abused alcohol and smoked [21]. A similar depletion of GSH and oxidation of the GSH/GSSG potential occurred in the exhaled breath condensate of subjects that abused alcohol suggesting oxidation throughout the respiratory tree [22]. 

**Figure 2:**
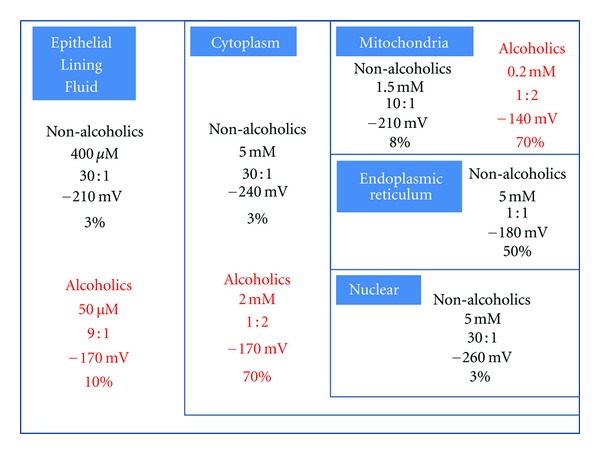
GSH/GSSG redox potentials in extracellular and intracellular GSH pools. Values indicate the absolute GSH concentration (from *μ*M to mM), the ratio of GSH and GSSG, GSH/GSSG redox potential (mV), and % of GSSG (calculated as the ratio of GSSG concentration over total both reduced and oxidized (GSH + GSSG) concentration). Values were taken from [19–22, 24, 25].

For alveolar macrophages (AM), ELF is the only source of GSH and AM cellular functions are dependent on this GSH availability [23]. In the ethanol-fed rat model, the depletion and oxidation of the GSH pool in the ELF were mirrored in the AM with a similar decrease in GSH and ~30 mV oxidation of the GSH/GSSG [24]. In alveolar type II (ATII) cells, there was a 60% decrease in GSH and ~40 mV oxidation in GSH/GSSG in cells derived from ethanol-fed rats [25]. This ethanol-induced oxidation was also reflected in organelles where there was an ~60 mV oxidation in GSH/GSSG redox potential in the mitochondria from ATII cells [25]. A scheme for GSH/GSSG redox potentials in extracellular and intracellular GSH pools is shown in Figure 2. It is interesting to note that across intracellular and extracellular GSH pools in alveolar type II cells and alveolar macrophages, chronic alcohol ingestion consistently induced a 30–60 mV oxidation of GSH/GSSG suggesting the redox potential of different alveolar GSH pools are in equilibrium. 

**Figure 3:**
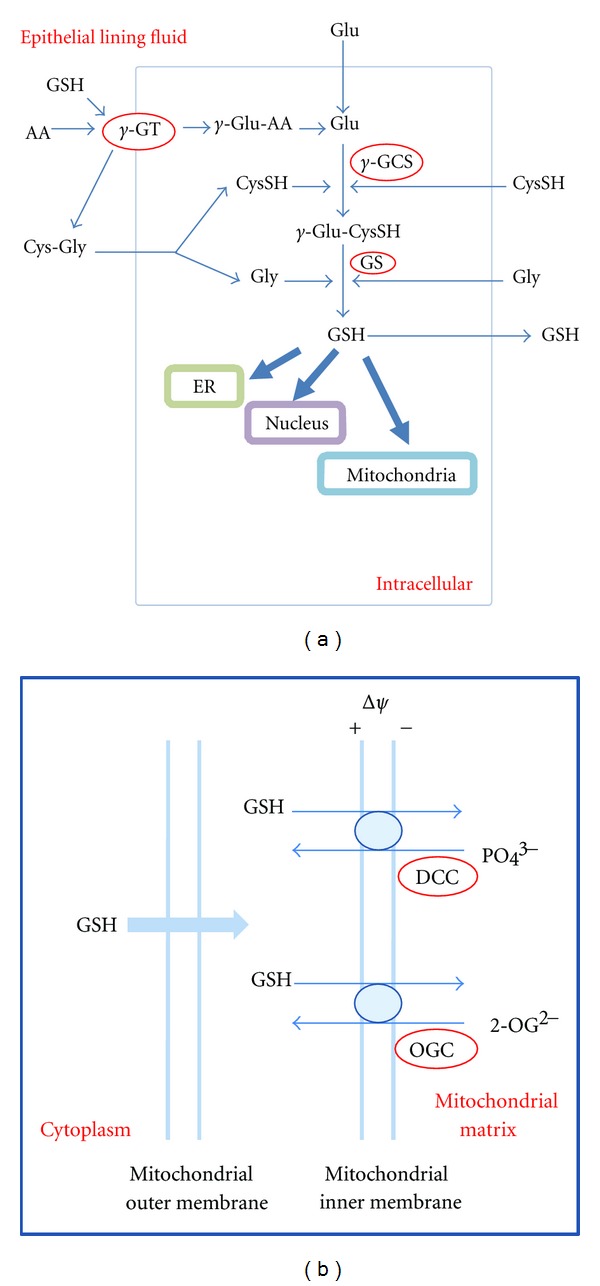
Glutathione synthesis, transportation, and compartmentalization. (a) GSH *de novo* synthesis requires glutamate (Glu), cysteine (Cys), and glycine (Gly). Key enzymes involved are g-GT: g-glutamyltransferase; g-GCS: *γ*-glutamylcysteinesynthetase; GS: glutathionsynthetase. GSH is freely distributed in the cytosol, but also compartmentalized in mitochondria, nucleus and ER. (b) GSH transports from cytosol to mitochondrion. GSH can move easily through the mitochondrial outer membrane, but requires carrier-mediated transport to cross the mitochondrial inner membrane. DCC: dicarboxylate carrier; OGC: 2-oxoglutarate carrier.

GSH is unique among thiol-based antioxidants in that it is only a tripeptide composed of glutamate (Glu), cysteine (Cys), and glycine (Gly). However, it possesses an unusual peptide bond in that cysteine and glutamate are linked through the *γ*-carboxyl group of glutamate, instead of the normal *α*-carboxyl group. GSH is synthesized in two steps (Figure 3). In the initial step, *γ*-glutamylcysteine synthetase (*γ*-GCS) forms a peptide bond between the Glu *γ*-carboxyl group and the Cys amino group using the energy provided by one ATP. In the following step, glutathione synthetase (GS) forms a peptide bond between Gly and *γ*-Glu-CysSH by consuming a second ATP. The first step, catalyzed by *γ*-GCS, is rate limiting since *γ*-GCS activity is regulated by a negative feedback mechanism for GSH. Given that GSH synthesis depends on the energy provided by ATP and alcohol is associated with decreased ATP generation, decreased ATP availability could ultimately lead to decreased GSH synthesis [26, 27]. For GSH, its *γ*-carboxyl peptide bond protects against hydrolyzation by most peptidases that catalyze the cleavage of *α*-carboxyl peptide bonds. Rather, the *γ*-carboxyl peptide bond is cleaved by *γ*-glutamyltransferase (*γ*-GT) on the external surface of certain cell types (Figure 3). This cleavage produces *γ*-glutamyl amino acid (*γ*-Glu-AA), which is further transformed to yield Glu for *de novo* GSH synthesis. In addition, the C-terminus Gly residue prevents the cleavage by intracellular *γ*-glutamylcyclotransferase. Therefore, GSH is relatively stable and is only cleaved at the outer membrane of certain cells.

GSH has the ability to scavenge both reactive oxygen species (ROS) and reactive nitrogen species (RNS). Upon oxidation, two hydrogens are donated to form the GSH disulfide (GSSG). Glutathione reductase (GR) reduces GSSG back to GSH using NADPH as the electron donor. Thus, chronic alcohol ingestion can affect GSH homeostasis through (1) alcohol-induced generation of ROS and RNS which promote GSH oxidation, and (2) imbalanced ratios of NAD^+^/NADH and NADP^+^/NADPH which attenuate GSSG reduction [19, 24, 28]. Therefore, the consequence of chronic alcohol ingestion is GSSG accumulation. Alcohol abuse may also impair GSH transport. For ATII cells and AM, the intracellular GSH concentrations are ~5 mM in contrast to ~400 *μ*M in the ELF, representing only a 10-fold difference. Intracellularly, the majority of GSH (about 90%) is freely distributed in the cytosol, but can also be found compartmentalized in organelles such as mitochondria, nuclei, and endoplasmic reticulum (ER).

Alcohol-induced depletion and oxidation of GSH suggests that alcohol promotes ROS generation through multiple mechanisms and decreases the capacity of the normal cellular defense mechanisms to protect against those ROS. Molecular oxygen is essential for cellular functions because it plays a pivotal role in mitochondrial ATP synthesis, the cellular energy source. However, chronic alcohol abuse has long been known to depress mitochondrial structure and function which contribute to decreased respiration, increased mitochondrial ROS, decreased ATP, and disrupted fatty acid metabolism [29]. Within the mitochondria, NADH is oxidized to NAD^+^ during which molecular oxygen is the electron acceptor and NADH is the electron donor. In the mitochondrial electron transport chain, oxidation reactions remove a hydrogen/proton or an electron from a molecule through the proton pumps complexes I, III, IV and these reactions are coupled to the mobile electron carrier coenzymes ubiquinone (Q) and cytochrome c. This series of electron transfer reactions are necessary to synthesize ATP. However, different oxygen radicals can form intermediate products that are considered primary ROS, including superoxide anion (O_2_^•−^), peroxide ion (O_2_^2−^) which forms hydrogen peroxide (H_2_O_2_), and hydroxyl radical (^•^OH) [30–32].

Alcohol metabolism involves alcohol dehydrogenase (ADH) which converts ethanol to acetaldehyde and mitochondrial aldehyde dehydrogenase (ALDH) which converts acetaldehyde to acetate in mitochondria [33, 34]. Both ADH and ALDH use the cofactor NAD^+^ which is reduced to NADH. With alcohol-induced changes in the cellular NAD^+^/NADH ratio, there is an accompanying impairment in ATP synthesis. As a result, the uncoupling of the electron transport chain increases mitochondrial generation of superoxide anion (O_2_^•−^), H_2_O_2_, and hydroxyl radical (^•^OH). Acetaldehyde, a product of alcohol metabolism, can react with proteins and lipids and lead to free radical formation. Alcohol also induces the enzyme cytochrome P450 2E1 (CYP2E1) activity, which metabolizes alcohol and generates ROS. Alcohol metabolism also promotes the generation of alcohol-derived radicals, such as 1-hydroxyethyl radical [35–37]. Moreover, in lung tissue and AMs, alcohol also upregulates the expression and activities of NADPH oxidase isoforms, which use molecular oxygen and NADPH to generate ROS [38, 39]. 

In addition to ROS generation, alcohol can also interfere with the cellular antioxidant defense system [23, 40]. In the mitochondria, NADPH-dependent thiol-disulfide redox molecules such as GSH, glutaredoxin 2 (Grx2), thioredoxin 2 (Trx2), and peroxiredoxin 3 (Prdx3) regulate the activities of redox-sensitive proteins containing cysteines. This makes these thiol/disulfide switches key components of low-flux redox circuits in cell signaling and control of metabolic redox [41, 42]. NADPH is primarily produced by nicotinamide nucleotide transhydrogenase (NNT) which uses the mitochondrial proton gradient to transfer protons between NADH and NADP^+^. As described above, alcohol metabolism changes the ratio of NAD^+^/NADH which can affect the cellular NADPH state. However, these thiol-disulfide redox switches depend on NADPH and modified NADPH availability decreases the defensive capacity of this mitochondrial thiol-disulfide system.

GSH compartmentalization within different organelles is regulated through specific transport mechanisms. The mitochondrial GSH pool is the main line of defense for maintaining the redox environment of the thiol/disulfide switches mentioned above as well as the redox environment of the mitochondria. Therefore, maintenance of the GSH pool in the mitochondria will prevent or reverse oxidative modifications. GSH can cross the mitochondrial outer membrane through porin channels and then is transported across the mitochondrial inner membrane into the mitochondrial matrix through the 2-oxoglutarate carrier (OGC) and the dicarboxylate carrier (DCC) [43] (Figure 3). The end result is that the mitochondria have a GSH concentration similar to the cytosol [44, 45]. Within the mitochondria, GSH detoxifies hydrogen peroxides, lipid hydroperoxides, or xenobiotics, mainly as a cofactor for enzymes such as glutathione peroxidases. For ER, GSH is transported across the ER membrane through protein facilitated diffusion in the ER membrane. Unlike the cytosol and mitochondria, GSH exists mainly as GSSG which plays a central role in disulfide bond formation for the folding of nascent proteins in the ER [46, 47]. The mechanism for nuclear GSH transport is unclear and may rely on passive diffusion of GSH from the cytosol into the nucleus via nuclear pores. The nuclear GSH pool is directly correlated with cell cycle progression. In the proliferative state, nuclear GSH is higher than cytosolic GSH, while GSH is equally distributed between the cytosol and nuclear compartment when cells become confluent. 

**Figure 4:**
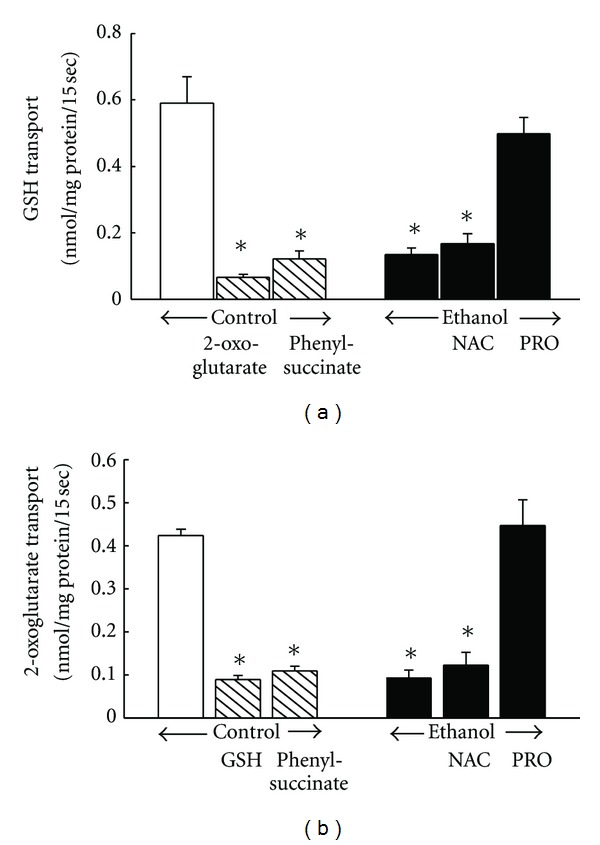
Ethanol impaired mitochondrial GSH (a) and 2-oxoglutarate (b) uptake. The investigation of mitochondrial uptake of GSH and 2-oxoglutarate revealed that procysteine (PRO) but not N-acetylcysteine (NAC) protected the 2-oxoglutarate transporter during ethanol ingestion. The uptakes of ^3^H-GSH and ^14^C-oxoglutarate were assessed in mitochondria isolated from alveolar type II cells from rats fed control, ethanol, ethanol + NAC, or ethanol + PRO diets. (*P ≤ 0.05) compared to controls.

The steady state level of intracellular and extracellular GSH is well-balanced by production, consumption, and transport [48]. Alcohol-induced GSH depletions in the mitochondria and the ER have been demonstrated in hepatocytes [49, 50]. In previous studies with a rat model, we demonstrated that chronic ethanol ingestion decreased the cellular GSH pool of ATII cells and this was associated with decreased cellular functions such as surfactant synthesis and secretion as well as viability [25]. Furthermore, the addition of the GSH precursors S-adenosyl-L-methionine and N-acetylcysteine to the diet during the final week of ethanol ingestion significantly reduced the risk of lung injury suggesting that GSH depletion predisposes the lung to acute lung injury after endotoxemia. As noted above, chronic ethanol ingestion also decreased the mitochondrial GSH pool in ATII cells by 80% with an accompanying oxidation of the GSH/GSSG redox potential by ~60 mV. However, restoration of the mitochondrial GSH pool with the GSH precursor, procysteine, during the last week of ethanol ingestion rescued surfactant synthesis and secretion [51]. In addition to increased GSH utilization, one study from our lab indicated that depletion of the mitochondrial GSH pool was due to ethanol inhibition of mitochondrial GSH uptake by the 2-oxoglutarate carrier (OGC) (Figure 4). Procysteine reversed ethanol-induced OGC inhibition and rescued the mitochondrial GSH pool. Additional studies are needed to determine if chronic ethanol ingestion promotes a similar depletion of GSH or oxidation of the GSH/GSSG redox potential in other organelles of the ATII cell or other cell types. 

## 3. Chronic Alcohol Abuse and Pulmonary Immune Function: Oxidative-Stress-Induced Immune Dysfunction

Both acute and chronic alcohol consumption have well-documented effects on the immune system leading to increased susceptibility to community acquired pneumonia and tuberculosis [52, 53]. In the presence of an alcohol use disorder, subjects with pneumonia are more likely to be infected with a serious Gram-negative bacteria such as *Klebsiella pneumoniae *[54], have a greater likelihood that the pneumonia is invasive [55], and that the bacteria has acquired antimicrobial resistance [56]. These increased risks occur even in those who do not meet the diagnostic criteria for an alcohol use disorder [57]. As noted above, the outcome when a septic patient has a history of alcohol abuse is poorer with greater mortality and greater health care costs. There is also an increased risk of ventilator-associated pneumonia which worsens the morbidity and mortality rates in critically ill patients [58–61]. 

Although it is well recognized that alcohol abuse impairs alveolar macrophage immune function and renders patients susceptible to pneumonia, the mechanisms involved are incompletely understood. In the alveolar space, AMs phagocytose microbes and orchestrate the immune response, but alcohol abuse suppresses immune responses, such as the release of TNF-*α* and phagocytic capacity [24, 39, 62–65]. Salient to alcohol-induced immunosuppression is the chronic oxidative stress [39] which may be related to GSH depletion and GSH/GSSG oxidation in the ELF [24, 66]. The ELF pool of GSH is an essential source for AM uptake of GSH which is required for protection from oxidant injury and maintenance of membrane integrity during a respiratory burst. Indeed, the GSH/GSSG redox potential of AM derived from ethanol-fed rats is oxidized ~30 mV, similar to that observed in the ELF. When GSH availability in the AM is limited, cellular functions such as phagocytosis and microbial clearance are compromised. Similarly, alcohol mediated decreases in GSH availability and GSH/GSSG oxidation are associated with accompanying increases in AM apoptosis [24]. However, restoration of the GSH pool and the GSH/GSSG redox potential of the ELF restore the phagocytic capacity of the AM [24, 66] further supporting the suggestion that the GSH/GSSG redox state of the ELF modulates AM immune responses. Indeed, restoration of the GSH pool in the ELF through oral GSH precursors decreased the risk of respiratory infections in the ethanol-fed rat model [67]. Similarly, fetal ethanol exposure impairs the phagocytic function of the newborn AM but maternal diets containing GSH precursors or intranasal delivery of GSH to the newborn pup restored AM phagocytosis and decreased risk of respiratory infections [68, 69].

The balance of intracellular oxidants and antioxidant systems is critical in the regulation of receptors and cytokines in the immune response. Alcohol induced ROS generation and GSH oxidation may alter the response of other immune cells. In contrast to the AM, oxidation of intracellular GSH and ROS induce the functional activation of T lymphocytes [70]. However, the AM phenotype also regulates the ratio of type 1 to type 2 (TH1/TH2) helper T cells [71]. When the intracellular GSH pool is decreased, the AM are referred to as “oxidative” macrophages and exhibit a TH2-dominant immune response. In contrast, AM with an elevated GSH pools are referred to as “reductive” macrophages which generate a TH1-type response. 

In macrophages, an alternatively activated phenotype, also known as M2 activation, is additionally associated with TH2 anti-inflammatory processes and suppression of phagocytosis. With chronic ethanol ingestion, there is increased expression of the immunosuppressive TGF*β*_1_ and IL-13 which establish an autocrine feed forward loop in the AM [65]. Since TGF-*β*_1_ and IL-13 are markers for alternative activation, this switch to a M2 phenotype may contribute to the alcohol-induced suppression of phagocytosis. Indeed, inhibition of TGF*β*_1_ or IL-13 signaling rescued phagocytosis in the AM derived from ethanol-fed mice. Generally, alternative activation works toward resolution of inflammation and promotion of wound repair. However, alcohol-induced chronic stimulation of TGF-*β*_1_ and IL-13 signaling eventually leads to chronic suppression of phagocytosis by the AM, thereby increasing the risk of respiratory infections.

Granulocyte/macrophage colony-stimulating factor (GM-CSF) is a trophic factor for the alveolar epithelium which secretes GM-CSF into the ELF where it is required for the priming of ATII cells. In ATII cells derived from ethanol-fed rats, there is decreased expression of GM-CSF receptors and this is associated with loss of barrier integrity [63, 72]. In the endotoxemia model, *in vivo* treatments with GM-CSF dramatically improved alveolar epithelial barrier integrity, particularly in the ethanol-fed rat. This suggested that GM-CSF has previously unrecognized effects in promoting alveolar epithelial barrier integrity and that these salutary effects may be particularly relevant in the setting of chronic alcohol abuse.

For immune cells, GM-CSF stimulates the production of granulocytes and monocytes, their migration into tissue, and their maturation into AM and dendritic cells. However, chronic alcohol ingestion also decreases the expression of GM-CSF receptors in AM [72]. In parallel, ethanol ingestion decreased cellular expression and nuclear binding of PU.1, the master transcription factor that activates GM-CSF-dependent AM functions. Thus, the impaired terminal differentiation of AM associated with chronic ethanol ingestion may be a consequence of alcohol-induced decreases in the expression of GM-CSF receptors and PU.1. This is further supported by the observation that treatment of the ethanol-fed rat with recombinant GM-CSF restored GM-CSF receptor expression and signaling as well as AM differentiation and accompanying immune functions [72]. Whether GM-CSF treatments of the ethanol-fed rat restore GSH pools and attenuate oxidative stress remains to be determined. However, treatment of the ethanol-fed rat with GSH precursors restores GM-CSF and PU.1 signaling suggesting another cascade of events by which alcohol-induced oxidant stress impairs AM functions.

## 4. Conclusion and Perspectives

Chronic ethanol ingestion induced GSH depletion and oxidation in the exhaled breath condensate, the ELF, as well as the intracellular compartments of ATII cells and AM. This GSH depletion and oxidation resulted in chronic oxidative stress as evidenced by a 40–60 mV GSH/GSSG oxidation in all the GSH pools tested. This oxidant stress was associated with impaired functions of ATII cells such as decreased surfactant processing, decreased epithelial barrier integrity, and increased risk of apoptosis. For the AM, this oxidative stress was associated with impaired terminal differentiation, alternative activation, impaired phagocytosis, and an increased risk of apoptosis. This compromise in ATII cell and AM cellular functions may explain the increased risk of respiratory infections and risk of developing ARDS in subjects with an alcohol use disorder. Strategies to decrease alcohol abuse are clearly needed. However, increased risks of bacterial pneumonia and tuberculosis are hallmarks of alcohol abuse, even in those not meeting the clinical definition of an alcohol abuse disorder. Therefore, understanding the mechanisms by which alcohol-induced oxidation of the GSH/GSSG redox potential in the ELF leads to compromised functions of ATII cells and AM are clearly needed in order to decrease the incidence of alcohol-related pneumonia and ARDS as well as their associated healthcare costs. Failure to understand the underlying mechanisms by which alcohol abuse or chronic oxidative stress impairs the contributions of AM to the alveolar immune response not only contributes to increased morbidity and mortality in this vulnerable population but also increases the risk of dissemination of novel strains of influenza, bacterial pneumonia, and rifampicin resistant tuberculosis [73].
